# Associations of sarcopenia, sarcopenia parameters and motoric cognitive risk syndrome in Chinese older adults

**DOI:** 10.3389/fnagi.2023.1302879

**Published:** 2023-11-22

**Authors:** Dian Jiang, Xi Chen, Jundan Huang, Lina Wu, Yifei Chen, Hui Feng, Mingyue Hu

**Affiliations:** ^1^Xiangya School of Nursing, Central South University, Changsha, Hunan, China; ^2^Xiangya-Oceanwide Health Management Research Institute, Central South University, Changsha, Hunan, China; ^3^National Clinical Research Center for Geriatric Disorders, Xiangya Hospital of Central South University, Changsha, China

**Keywords:** motoric cognitive risk syndrome, sarcopenia, sarcopenia parameters, five-times sit-to-stand test, handgrip strength, older adults

## Abstract

**Background:**

Motoric cognitive risk syndrome (MCR) is a pre-dementia symptom strongly predicting cognitive decline and dementia. Although advancements in elucidating the epidemiology of MCR, the evidence about the association between sarcopenia, sarcopenia parameters, and MCR remains scarce.

**Objectives:**

The purpose of this study was to determine the associations between sarcopenia, sarcopenia parameters, and MCR among community-dwelling Chinese older adults.

**Methods:**

A total of 4,184 community-dwelling older adults from the China Health and Retirement Longitudinal Study (CHARLS) in the 2011 waves were included. Sarcopenia was diagnosed according to the Asian Working Group for Sarcopenia criteria. Sarcopenia parameters included handgrip strength (HGS), height-adjusted appendicular skeletal muscle mass (ASM/Ht^2^), and five-times sit-to-stand test time (FTSSTT). MCR was defined as subjective cognitive complaints and slow gait speed without dementia or impaired mobility. The associations between sarcopenia, sarcopenia parameters, and MCR were conducted using the logistic regression model. The restricted cubic spline with four knots were performed to determine the nonlinear and linear relationships between HGS, ASM/Ht^2^, FTSSTT, and MCR.

**Results:**

The prevalence of MCR in wave 2011 of CHARLS was 11.2%. After adjustment for potential confounders, we found sarcopenia [odd ratio (OR) (95% CI): 1.70 (1.13 ~ 2.54), *p* = 0.011], lower HGS [0.97 (0.96 ~ 0.99), *p* = 0.001], and more FTSSTT [1.12 (1.10 ~ 1.15), *p* < 0.001] were significantly associated with a higher risk of MCR. There was an inverse linear dose–response between HGS and MCR (*p* for overall = 0.008, p for nonlinearity =0.776). The nonlinear relationship between FTSSTT and MCR was found (*p* for overall <0.001, *p* for nonlinearity = 0.025) with FTSSTT ≥29 s being associated with a higher risk of MCR. A dose–response relationship was not found between ASM/Ht^2^ and MCR (*p* for overall =0.589).

**Conclusion:**

Sarcopenia, lower HGS, and higher FTSSTT are associated with MCR among older adults in China, while the latter two exhibit a dose–response relationship with MCR. It is suggested that timely identification and management of sarcopenia and its parameters may help delay the progression of cognitive impairment and promote healthy aging.

## 1 Introduction

With this rapid global expansion of the aging population, it is estimated that the number of people with dementia worldwide will increase from 57.4 million in 2019 to 152.8 million in 2050 (Collaborators, 2022). Given no remarkable effective therapies for dementia, it could impose a heavy socio-economic burden (Tochel et al., 2019), especially in China, which has the highest number of patients with dementia (Jia et al., 2020). Motoric cognitive risk syndrome (MCR) is a pre-dementia symptom characterized by subjective cognitive impairment and slow gait speed (Verghese et al., 2014), two critical predictors of cognitive impairment and dementia (Mitchell et al., 2014; Peel et al., 2019). MCR strongly predicts cognitive decline and dementia (Liu et al., 2021; Mullin et al., 2022), and the risk is higher than for either slow gait speed or subjective memory complaint alone (Verghese et al., 2014). Meanwhile, MCR is associated with other detrimental health effects (Beauchet et al., 2019; Bai et al., 2022; Mullin et al., 2022), including increased mortality, disability, and falls. Establishing a clearer understanding of the epidemiology of MCR is crucial to helping specify reasonable preventive and intervention strategies for cognitive impairment.

Sarcopenia is a severe geriatric syndrome, mainly defined by an age-related loss of muscle mass and muscle function (Chen et al., 2020). Substantial evidence (Abellan Van Kan et al., 2013; Peng et al., 2020; Beeri et al., 2021; Salinas-Rodríguez et al., 2021; Chen et al., 2022; Hu et al., 2022; Lin et al., 2023) indicates a significant association between sarcopenia and cognitive performance, for instance, cognitive impairment, mild cognitive impairment, and Alzheimer’s disease, which is independent of the focusing population and region. Knowledge about the association between sarcopenia, sarcopenia parameters, and MCR is scarce and ambiguous. After adjusting for the potential covariates, only two studies (Zhang et al., 2022; Merchant et al., 2023) show a significant association between sarcopenia and MCR in older adults and pre-frail older adults. However, the evidence could be stronger with scientific sampling methods and larger sample size. Moreover, with the trend of highlighting the effect of muscle function on sarcopenia diagnosis (Cruz-Jentoft and Sayer, 2019), sarcopenia parameters attract more and more researchers’ attention. Birong (Jia et al., 2023) shows that handgrip strength (HGS) weakness is associated with MCR incidence. Nevertheless, the relationship between sarcopenia and MCR still needs to be verified by large-scale studies. Furthermore, few studies examine the dose–response relationship between sarcopenia parameters and MCR, which avoids masking or weakening significant associations due to the unknown shape of relational features.

To fill these gaps, we first conducted cross-sectional analyses to investigate the association between sarcopenia and MCR in the middle-aged and older Chinese population using the nationally representative data from the China Health and Retirement Longitudinal Study (CHARLS). Then, we aimed to determine the potential dose–response relationships between sarcopenia parameters, including HGS, height-adjusted appendicular skeletal muscle mass (ASM/Ht^2^), five-times sit-to-stand test time (FTSSTT), and MCR by the cubic spline analysis.

## 2 Materials and methods

### 2.1 Participants and procedures

We utilized data from the CHARLS, a nationally representative longitudinal study of Chinese adults aged 45 and above. The baseline national census of CHARLS was fielded in 2011, including about 10,000 households from 17,500 individuals in 150 counties/districts and 450 villages/resident committees through multistage stratified probability-proportionate-to-size sampling. The individuals were followed up every 2 years by a face-to-face computer-assisted personal interview. More details about objectives, design, and methods can be found in a previous study (Zhao et al., 2014).

**Figure 1:**
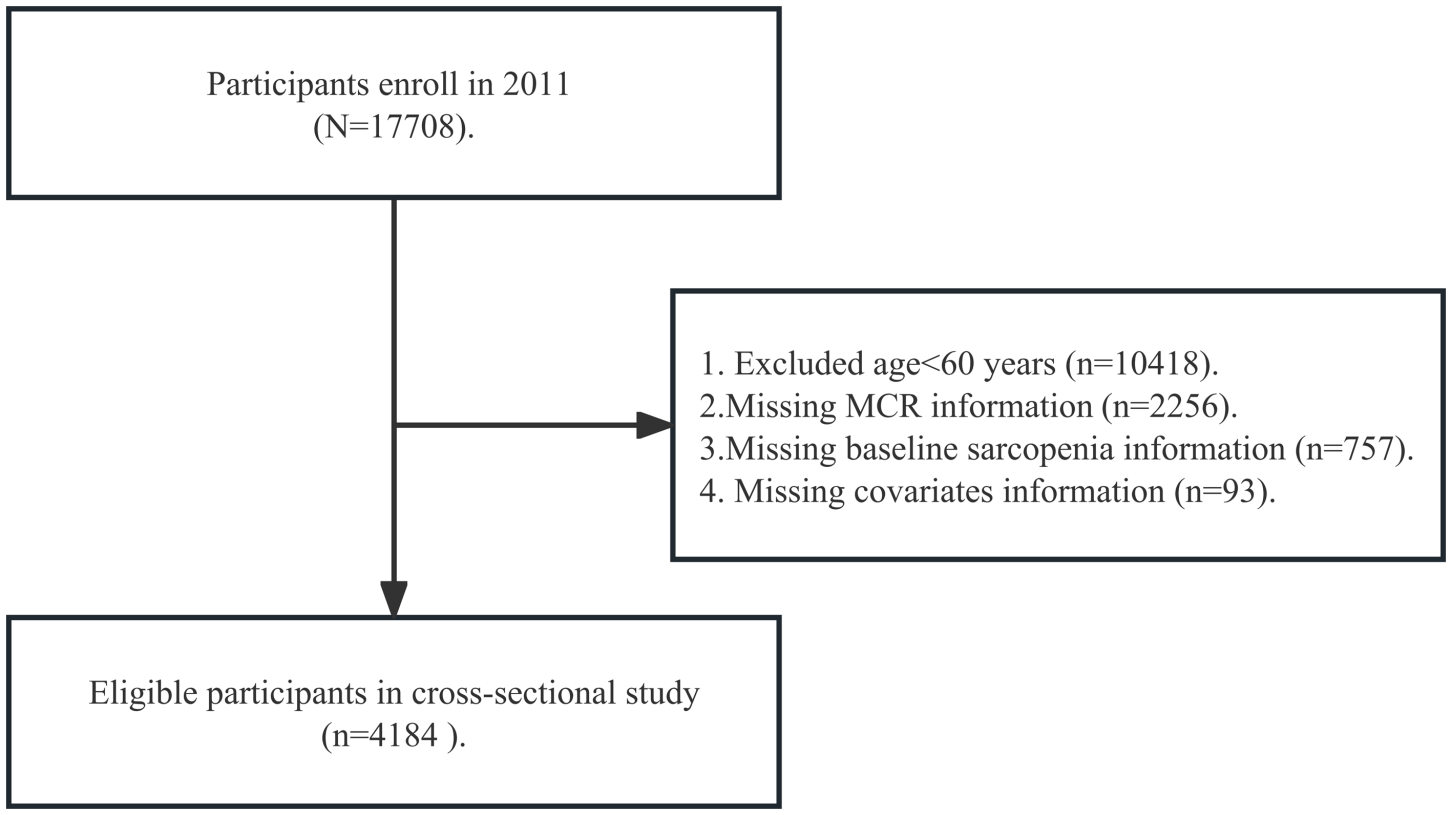
Flow diagram for participants included in the study. MCR, motoric cognitive risk syndrome.

We analyzed data from the CHARLS wave 2011, which was more valid and representative because partial participants in other waves were non-response and died. A total of 4,184 participants have been included from wave 2011. We included older individuals for the following reasons: (1) age ≥ 60 years old (participants aged 45 – 59 had not been included because most of them skipped the walking speed tests in CHARLS); (2) completing MCR information in baseline; (3) completing sarcopenia information in the baseline; (4) completing covariates in the baseline. Figure 1 shows the details of the participant selection procedure.

### 2.2 Measures

#### 2.2.1 Sarcopenia and sarcopenia parameters

According to the diagnostic criteria recommended by the Asian Working Group for Sarcopenia (AWGS) in 2019, sarcopenia was defined as low muscle mass plus low muscle strength or physical performance (Chen et al., 2020). Sarcopenia parameters in our study included HGS, ASM/Ht^2^, and FTSSTT. HGS (kg) was measured by the participant squeezing a Yuejian TM WL-1000 dynamometer twice as hard as possible by holding the dynamometer at a right angle (90°) (Zhao et al., 2014). HGS was the maximum measurement of the dominant hand. When the dominant hand could not be measured under particular circumstances, it used the maximum measurement of the other hand instead. HGS <28.0 kg for men and < 18.0 kg for women were considered low muscle strength (Chen et al., 2020). The muscle mass was estimated at ASM/Ht^2^ (kg/m^2^). Appendicular skeletal muscle mass using a validated anthropometric equation in Chinese residents (Wen et al., 2011). The agreement between the skeletal muscle mass equation model and dual X-ray absorptiometry was strong (Wen et al., 2011; Yang et al., 2017). The cut-off for defining low muscle mass was based on the sex-specific lowest 20% of the ASM/Ht^2^ among the study population (Gao et al., 2022). Therefore, low muscle mass refers to the ASM/Ht^2^ < 4.90 kg/m^2^ in women and < 6.79 kg/m^2^ in men. According to AWGS 2019, low physical performance was based on the Short-Physical Performance Battery, 6-m gait speed test, or FTSSTT. The diagnosis of MCR already contained a slow gait. To avoid the overlapping effect of gait speed on the relationship between sarcopenia and MCR, we used FTSSTT (seconds) as the index of physical function in sarcopenia. The FTSSTT ≥12 s is the poor physical performance cut-off value (Chen et al., 2020). Further details about the definitions for sarcopenia components in the CHARLS have been described previously (Wu et al., 2021).

#### 2.2.2 MCR

MCR syndrome was defined as cognitive complaints and slow gait speed without dementia or impaired mobility. Our study assessed cognitive complaints using a self-reported questionnaire about memory loss: “How would you rate your memory at present?” Respondents were asked to rank their answers on a scale of five: “excellent,” “very good,” “good,” “fair,” and “poor.” Those who reported “fair” or “poor” were recorded as having cognitive complaints. Gait speed was used to assess physical performance, computed by the average time respondents took to walk along a straight 2.5 m path twice. The cut-off slow gait speed was 1.0 standard deviations or below age (60–74, ≥75 years old) and sex (male and female) appropriate mean values of gait speed in our cohort (Verghese et al., 2014) (Supplementary Table S1).

#### 2.2.3 Covariates

All covariates were obtained at baseline. We included covariates associated with sarcopenia or MCR reported in previous research (Xu et al., 2021; Iqbal et al., 2022; Yuan and Larsson, 2023). Sociodemographic characteristics included in the analysis were as follows: age, gender (male/female), educational level (less than lower secondary education/secondary or above), marital status [single (divorced/widowed/single)/partnered (married/partnered)], and residence (rural region/urban region, defined by the National Bureau of Statistics of the People’s Republic of China). Lifestyle factors included smoking history (former or non-smokers/current smokers, self-reported), alcohol consumption (yes/no consumed any alcohol last year, self-reported), and self-reported sleep duration. Health indicators included a self-reported history of common co-morbidities (hypertension, diabetes heart disease, and stroke), depression measured by the 10-item Center for Epidemiological Studies Depression Scale (CES-D), body mass index (BMI) calculated as weight in kilograms divided by the square of height in meters, falls assessed by a self-reported by a question, “Have you fallen down in the last 2 years?”

### 2.3 Statistical analyzes

Characteristics of participants were presented as means ± standard deviations for continuous and frequency with percentage for categorical variables, respectively. The normal distribution was assessed by histogram. Differences between participants with and without MCR were compared using independent sample t-tests, the Mann–Whitney U test for continuous variables, and the chi-square test for categorical variables.

Logistic regression models were applied to estimate the association between sarcopenia, sarcopenia parameters, and MCR. Four models were fitted. Model 1 adjusted for age and gender. Model 2 adjusted for the covariates in Model 1 plus marital status, residence, education, smoking status, drinking status, and sleep time. Model 3 adjusted for the covariates in Model 2 plus hypertension, diabetes, heart disease, stroke, CES-D scores, and fall. Model 4 adjusted for the covariates in Model 3 plus BMI.

We further analyzed the dose–response relationships between sarcopenia parameters, including HGS, ASM/Ht^2^, FTSSTT, and MCR. The nonlinearity test was performed using the analysis of variance (ANOVA). Nonlinear relationships were defined as p lower than 0.05. The restricted cubic spline with four knots was performed to determine the nonlinear and linear relationships between HGS, ASM/Ht^2^, FTSSTT, and MCR. The confounders (age, gender, education, marital status, residence, alcohol consumption, smoking history, BMI, sleep, fall, CES-D scores, hypertension, diabetes, heart disease, and stroke) were considered in the model.

All statistical tests were represented as the odd ratio (OR) with 95% confidence interval (CI). All analyzes were conducted using IBM SPSS version 26.0 and R statistical software.

## 3 Results

### 3.1 Characteristics of the participants

Among 4,148 participants, 463 (11.2%) individuals had MCR, 3490 (83.4%) individuals had subjective cognitive complaint, 533 (13.2%) individuals had gait slowing and 604 (14.6%) individuals were absence of SCD and gait slowing. The mean age of study participants was 67.3 ± 6.1 years, and 47.7% were female.

The descriptive characteristics of the participants are shown in Table 1. Participants with MCR were more likely to be older (*p* = 0.006) and had a higher proportion of divorced/widowed/single (*p* = 0.010) than partnered. We found no difference between sex distribution and MCR status. Participants with MCR had slightly lower sleep time (*p* < 0.001), lower BMI scores (*p* = 0.011), and higher CES-D scores (*p* < 0.001). No significant difference in other sample characteristics was found between those with and without MCR. The prevalence of MCR in baseline participants was 16.4% (84/511) in sarcopenia and 10.3% (379/3673) in non-sarcopenia.

**Table 1:** Characteristics of all participants by MCR status.

	All (*n* = 4,184)	MCR (*n* = 463)	Non-MCR (*n* = 3,721)	*p* value
*Demographic*				
Age (years, M±SD)	67.3 ± 6.1	68 ± 6.3	67.2 ± 6.1	**0.006**
Gender (*n*, %)				0.834
Male	2,188 (52.3)	240 (51.8)	1,948 (52.4)	
Female	1,996 (47.7)	223 (48.2)	1,773 (47.6)	
Education (*n*, %)				0.056
Lower than secondary school	3,950 (94.4)	446 (96.3)	3,504 (94.2)	
Secondary or above	234 (5.6)	17 (3.7)	217 (5.8)	
Marital status (*n*, %)				**0.010**
Single	807 (19.3)	110 (23.8)	697 (18.7)	
Partnered	3,377 (80.7)	353 (76.2)	3,024 (81.3)	
Residence (*n*, %)				0.973
Urban areas	1,494 (35.7)	165 (35.6)	1,329 (35.7)	
Rural areas	2,690 (64.3)	298 (64.4)	2,392 (64.3)	
*Healthy lifestyles*				
Alcohol consumption (*n*, %)				0.158
No	2,834 (67.7)	327 (70.6)	2,507 (67.4)	
Yes	1,350 (32.3)	136 (29.4)	1,214 (32.6)	
Smoking history (*n*, %)				0.616
Current smokers	1,852 (44.3)	210 (45.4)	1,642 (44.1)	
Former or non-smokers	2,332 (55.7)	253 (54.6)	2,079 (55.9)	
Sleep (hours, M±SD)	6.2 ± 2.0	5.9 ± 2.1	6.2 ± 1.9	**<0.001**
Health indicators				
BMI (kg/m^2^, M±SD)	22.8 ± 4	22.4 ± 4.3	22.8 ± 4	**0.011**
CES-D (M±SD)	8.6 ± 6.2	10.1 ± 6.5	8.4 ± 6.2	**<0.001**
Hypertension (*n*, %)				0.114
No	2,855 (68.2)	301 (65.0)	2,554 (68.6)	
Yes	1,329 (31.8)	162 (35.0)	1,167 (31.4)	
Diabetes (*n*, %)				0.285
No	3,899 (93.2)	426 (92)	3,473 (93.3)	
Yes	285 (6.8)	37 (8.0)	248 (6.7)	
Heart disease (*n*, %)				0.832
No	3,557 (85.0)	392 (84.7)	3,165 (85.1)	
Yes	627 (15.0)	71 (15.3)	556 (14.9)	
Stroke (*n*, %)				0.112
No	4,069 (97.3)	445 (96.1)	3,624 (97.4)	
Yes	115 (2.7)	18 (3.9)	97 (2.6)	
Fall (*n*, %)				0.460
No	3,423 (81.8)	373 (80.6)	3,050 (82)	
Yes	761 (18.2)	90 (19.4)	671 (18)	
Sarcopenia (*n*, %)				**<0.001**
No	3,673 (87.8)	379 (81.9)	3,294 (88.5)	
Yes	511 (12.2)	84 (18.1)	427 (11.5)	

Bold values mean *p* < 0.05. MCR, motoric cognitive risk syndrome; BMI, body mass index; CES-D, Center for Epidemiological Studies Depression Scale; M±SD, mean ± standard deviation.

### 3.2 Association between sarcopenia, sarcopenia parameters, and MCR

**Figure 2:**
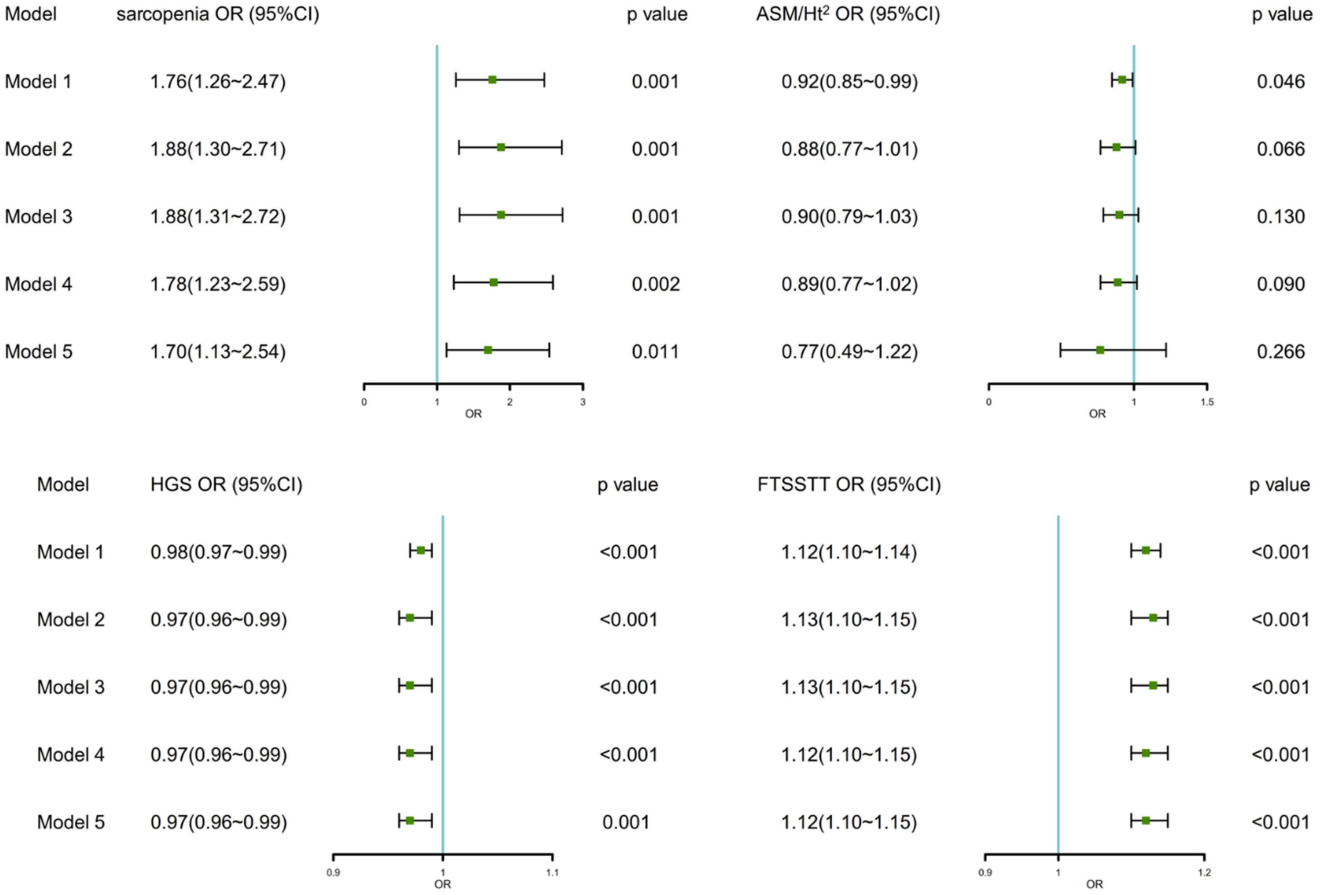
Associations between sarcopenia, sarcopenia parameters, and MCR. OR, odd ratio; CI, confidence interval; ASM/Ht^2^, height-adjusted appendicular skeletal muscle mass; HGS, handgrip strength; FTSSTT, five-times sit-to-stand test time. Model 1: No adjustment; Model 2: Adjusted for age, gender, residential area, education, and marital status; Model 3: Model 2 + ever/current smoke, ever/current alcohol, and daily sleep time; Model 4: Model 3 + co-morbidities, CES-D scores, and fall; Model 5: Model 4 + BMI.

Figure 2 shows the associations between sarcopenia, sarcopenia parameters, and MCR in logistic regression analyzes. We found significant associations between sarcopenia, HGS, FTSSTT, and MCR in crude and fully adjusted models. After controlling all covariates, sarcopenia was related with 1.70 times (95% CI:1.13 ~ 2.54) higher odds of MCR, and each 1 kg decrease in the HGS increased the odds of MCR by 3% (95% CI:1% ~ 4%) and each 1 s increase FTSSTT increased the odds of MCR by 12% (95% CI,10% ~ 15%). As for ASM/Ht^2^, although it was significantly related to MCR in the crude model, it was not significantly associated with MCR [0.88 (0.77 ~ 1.01), *p* = 0.066] after adjusting for demographics covariates.

### 3.3 Dose–response associations between sarcopenia parameters and MCR

**Figure 3:**
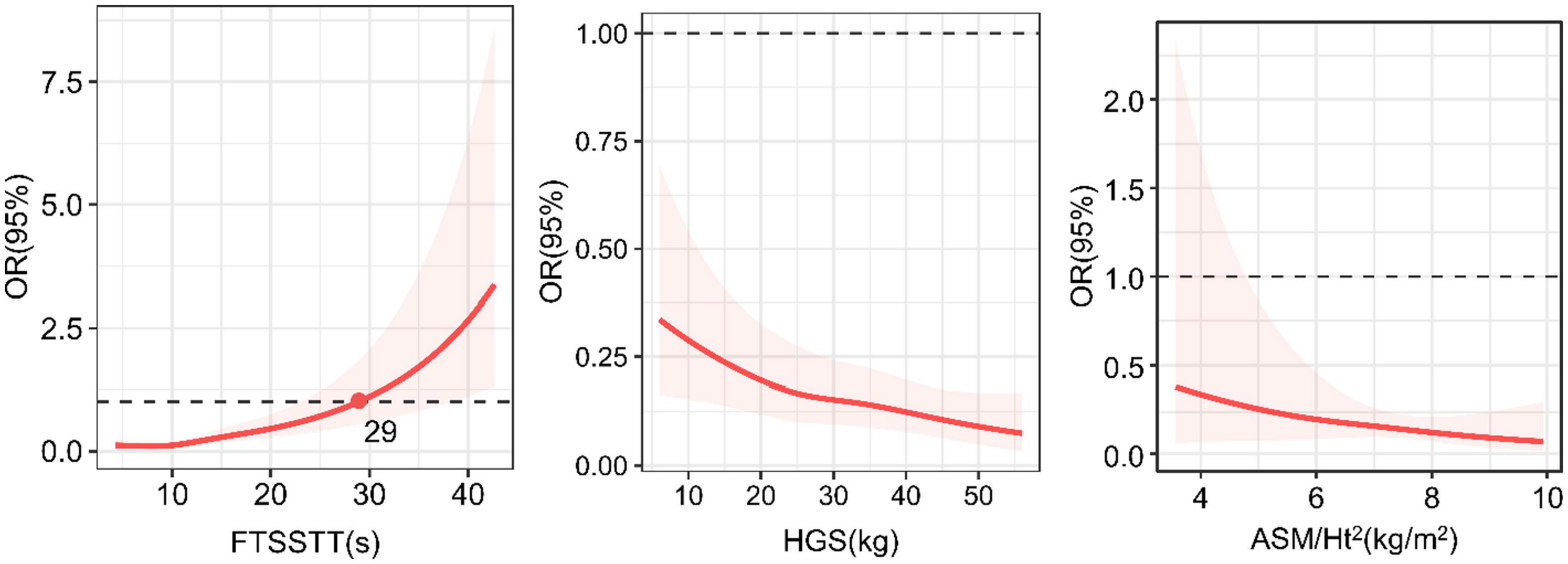
Dose–response relationships of sarcopenia parameters with motoric cognitive risk syndrome. OR, odd ratio; ASM/Ht^2^, height-adjusted appendicular skeletal muscle mass; HGS, handgrip strength; FTSSTT, five-times sit-to-stand test time.

Figure 3 shows the dose–response relationships of sarcopenia parameters with MCR. Regarding the HGS, we found an inverse linear dose–response relationship between HGS and MCR (p for overall = 0.008, p for nonlinearity =0.7775). Regarding the ASM/Ht^2^, we did not find a dose–response relationship between ASM/Ht^2^ and MCR (p for overall =0.589). Regarding the FTSSTT, we found a direct nonlinear dose–response association between the FTSSTT and MCR (p for overall <0.001, p for nonlinearity = 0.025), with the rate slowly at first and fast afterward. The FTSSTT is estimated at 29 s while the OR = 1.

## 4 Discussion

Based on nationally representative data of older Chinese adults, our study found that MCR was significantly associated with sarcopenia and the muscle function part of sarcopenia parameters, including HGS and FTSSTT. Results were still robust after controlling for the covariates associated with MCR and sarcopenia. Furthermore, we found a significant linear dose–response relationship between the HGS and MCR and a significant nonlinear dose–response relationship between the FTSSTT and MCR.

To our knowledge, this is the first large nationally representative study highlighting the significant association between MCR and sarcopenia, assessed by using the AWGS 2019 criteria. Our outcomes were consistent with previous studies conducted in 846 Chinese community-dwelling older adults (Zhang et al., 2022) and 397 pre-frail community-dwelling older adults (Merchant et al., 2023), reporting a significant cross-sectional relationship between sarcopenia and MCR, though the diagnostic criteria for sarcopenia and subjective cognitive decline differed. Further cohort studies were needed to confirm the causal relationship. Moreover, our study showed a significant relationship between muscle function and MCR rather than muscle mass after adjusting for covariates. This was consisting with evidence exploring relationships between sarcopenia parameters and cognitive impairment (Beeri et al., 2021; Bai et al., 2021b), which reveals that muscle function might be a more powerful predictor of cognitive performance than muscle mass alone, according to the trend of the definition of sarcopenia has shifted from skeletal muscle mass to strength and physical performance over the last two decades (Cruz-Jentoft and Sayer, 2019; Seino et al., 2022).

Importantly, we further evaluated the dose–response relationship between MCR and sarcopenia parameters. We found significant linear or nonlinear dose–response relationships between HGS, FTSSTT, and MCR, indicating more details and improving a deeper comprehension of the relationships. The increased odds of MCR linearly (SD) related to the decrease in HGS and nonlinear related to the increase in FTSSTT slowly at first and fast afterward. Precise trajectory shapes strengthened the robustness of significant associations between sarcopenia parameters and MCR. In addition, the cut-off point of FTSSTT was 29 s. While the value was above 29 s, the FTSSTT might be recognized as a risk factor for MCR. It is clinically significant to screen for MCR when the FTSSTT is 29 s and above.

The underlying mechanisms of sarcopenia involved in MCR have not been determined. It is suspected that both shared common risk factors (Beauchet et al., 2018; Gao et al., 2021; Stephan et al., 2021; Xu et al., 2021; Zeng et al., 2021; Sun et al., 2022), including age, smoking, physical inactivity, diabetes, heart disease, and sleep. Moreover, imbalanced secretion of endocrine and myokine cardiovascular factors are significantly associated with sarcopenia (Gao et al., 2022; Zuo et al., 2023) and MCR (Beauchet et al., 2018; Iqbal et al., 2022), suggesting vascular mechanisms may underlie the pathophysiology. Moreover, myokine imbalance caused by a reduction in skeletal muscle mass might harm neurons, and brain function may be the critical molecular mechanism (Jo et al., 2022; Mullin et al., 2022). Finally, dysregulation of the inflammatory pathway links sarcopenia and has been proven to mediate MCR in older adults (Michaud et al., 2013; Bai et al., 2021a). Future studies need to pay more attention to the mechanism of MCR.

There are several strengths of our study. Based on a large and nationally representative survey in China, our study made the results more generalized and reliable. In addition, to our knowledge, this study constitutes the first attempt to determine the dose–response relationships between sarcopenia parameters and MCR and clearly showed trajectories of the relationship between MCR and sarcopenia parameters. More importantly, our findings supported the validity of the primary algorithm of muscle function in sarcopenia diagnosis. Nevertheless, there are some limitations to this study. First, it is still potential that other confounding factors are omitted, although we have tried our best to include covariates based on prior knowledge as much as possible. Further, due to the limited sample of CHARLS, we did not investigate physical activity as an essential. Second, some people who did not complete the survey were excluded, potentially causing selection bias. Third, given the limitations of records available in the CHARLS, some variables were assessed by self-report (such as smoking history, alcohol consumption, sleep duration, falls, and co-morbidities), which might be subject to recall bias.

## 5 Conclusion

We demonstrated that sarcopenia, lower HGS, and higher FTSSTT are associated with higher MCR risk among Chinese adults aged 60 and above. Furthermore, significant inverse and linear dose–response relationships between HGS and MCR, as well as a significant direct nonlinear relationship between FTSSTT and MCR. Nevertheless, it is recommended that timely identification and management of sarcopenia and its parameters, particularly the FTSSTT, may be beneficial to prevent the incidence of MCR, delay the progression of cognitive impairment, and promote healthy aging.

## Data availability statement

Publicly available datasets were analyzed in this study. This data can be found at: http://charls.pku.edu.cn/en.

## Ethics statement

The studies involving humans were approved by the Biomedical Ethics Review Committee of Peking University (IRB00001052-11015). The studies were conducted in accordance with the local legislation and institutional requirements. The participants provided their written informed consent to participate in this study.

## Author contributions

DJ: Conceptualization, Formal analysis, Writing – original draft. XC: Conceptualization, Formal analysis, Writing – original draft. JH: Visualization, Writing – review & editing. LW: Writing – review & editing, Data curation. YC: Writing – review & editing, Data curation. HF: Conceptualization, Funding acquisition, Methodology, Supervision, Writing – review & editing. MH: Conceptualization, Funding acquisition, Methodology, Supervision, Writing – review & editing.
