# Supplementary material for: Associations of sarcopenia, sarcopenia parameters and motoric cognitive risk syndrome in Chinese older adults
Source: Front Aging Neurosci. 2023 Nov 22;15:1302879. doi: 10.3389/fnagi.2023.1302879 (PMC10703046; doi:10.3389/fnagi.2023.1302879)
Supplement: Supplementary file 1 [file Table_1.DOCX]

Supplementary Material

**Table S1. Cut-off for defining gait slowness.**

| Cut-off for defining slowness | |
| --- | --- |
| Men |  |
| age 60-74years | ≤45.37 cm/s |
| age ≥75 years | ≤35.10 cm/s |
| Women |  |
| age 60-74years | ≤40.70 cm/s |
| age ≥75 years | ≤28.93 cm/s |

**Table S2. Definition/codes of the variables.**

| Variable | Codes/definition |
| --- | --- |
| Sarcopenia and Sarcopenia Parameters | |
| Sarcopenia | 0=No; 1=Yes |
| HGS | Continuous variable |
| ASM/Ht^2^ | Continuous variable |
| FTSST | Continuous variable |
| Motoric Cognitive Risk Syndrome | |
| MCR | 0=No; 1=Yes |
| Covariates |  |
| Socio-demographics |  |
| Age | Continuous variable |
| Gender | 1=Male; 2=Female |
| Education | 1=Less than lower secondary education; 2=secondary or above |
| Marital status | 1=Single (divorced/widowed/single);  2=Partnered (married/partnered) |
| Residence | 1=Urban areas; 2= Rural areas |
| Health lifestyles |  |
| Alcohol consumption | Ever consumed any alcohol last year. 0=No; 1=Yes |
| Smoking history | Ever chewed tobacco, smoked a pipe, or smoked cigarette last year.  1=Current smokers; 0= former/non-smokers |
| Sleep | Self-reported sleep duration.  Continuous variable |
| Health Indicators |  |
| BMI | The weight in kilograms divided by the square of the height in meters.  Continuous variable |
| CES-D | Score of the 10-item Center for Epidemiological Studies Depression Scale (CES-D).  Continuous variable |
| Hypertension | 0=No; 1=Yes |
| Diabetes | 0=No; 1=Yes |
| Heart disease | 0=No; 1=Yes |
| Stroke | 0=No; 1=Yes |
| Fall | 0=No; 1=Yes |
